# The burden of chronic rhinosinusitis with nasal polyps and its relation to asthma in Finland

**DOI:** 10.1002/clt2.12200

**Published:** 2022-10-09

**Authors:** Sanna Toppila‐Salmi, Jenni Hällfors, Juhani Aakko, Bettina Mannerström, Kaisa Nieminen, Gunilla Telg, Lauri Lehtimäki

**Affiliations:** ^1^ Department of Allergology Department of Pulmonary Medicine, Heart and Lung Center Skin and Allergy Hospital Helsinki University Hospital and University of Helsinki Helsinki Finland; ^2^ Medaffcon Oy Espoo Finland; ^3^ AstraZeneca Nordic Espoo Finland; ^4^ AstraZeneca Nordic Södertälje Sweden; ^5^ Faculty of Medicine and Health Technology Tampere University Respiratory Research Group Tampere University Tampere Finland; ^6^ Allergy Centre Tampere University Hospital Tampere Finland

**Keywords:** asthma, comorbidity, CRSwNP, endoscopic sinus surgery, nasal polyps

## Abstract

**Background:**

Chronic rhinosinusitis with nasal polyps (CRSwNP) is commonly associated with asthma. Treatment of CRSwNP includes intranasal and systemic corticosteroids, with non‐responsive patients commonly considered for endoscopic sinus surgery (ESS). This nationwide register‐based study evaluated the incidence, prevalence, and treatment burden of CRSwNP in Finland, and their association with the presence and severity of comorbid asthma.

**Methods:**

Electronic health records of patients diagnosed with CRSwNP between 1.1.2012 and 31.12.2018 in Finnish specialty and primary care were included in the study. The patients were divided into subgroups based on presence, severity, and control of asthma: no asthma, mild to moderate asthma, severe controlled asthma, and severe uncontrolled asthma. A mean cumulative count of ESS was calculated over time per subgroup.

**Results:**

The prevalence of CRSwNP increased from 602.2 to 856.7 patients per 100,000 population between years 2012 and 2019 (*p* < 0.001). A total of 18,563 patients (59.9% male) had incident CRSwNP between 2012 and 2019, with 27% having asthma, 6% having severe asthma, and 1.5% having severe uncontrolled asthma. In the no asthma, severe controlled asthma, and severe uncontrolled asthma subgroups, systemic corticosteroids were used by 54.1%, 94.9% and 99.3% (*p* < 0.001), respectively, while the ESS count 3 years post diagnosis was 0.49, 0.68 and 0.80, respectively.

**Conclusions:**

The prevalence of CRSwNP showed a significant increase in the recent decade in Finland. Comorbid asthma, and in particular severe asthma, increased the probability of receiving systemic corticosteroids and undergoing ESS. Thus, improved management of CRSwNP in patients with comorbid asthma is urgently needed.

## 1 INTRODUCTION

Chronic rhinosinusitis (CRS) is a common disease with significant impact on the patients' health and the societal economy.^1^, ^2^ CRS is generally categorized into two major subtypes based upon phenotypic appearance: CRS without nasal polyps (CRSsNP) and CRS with nasal polyps (CRSwNP). Nasal polyps (NP) are benign inflammatory masses in the mucosa of the nose and paranasal sinuses.^1^, ^2^, ^3^ CRSwNP is associated with morbidity and decreased quality of life.^4^

CRS with nasal polyps is estimated to affect 1%–4% of the general population and 25%–30% of patients with CRS.^1^, ^4^ Yet, there is a paucity of prevalence data on CRSwNP across many geographic areas.^2^, ^5^, ^6^ Based on a few studies, the NP prevalence estimates in the Nordic countries range from 2.7% in Sweden to 4.3% in Finland, based on single municipality studies.^7^, ^8^ Subsequently, updated population‐based data on the prevalence and incidence of CRSwNP is required.

Epidemiological, clinical, and pathophysiological studies suggest that asthma is strongly associated with CRSwNP.^9^, ^10^ Inflammation in the nasal mucosa and lower airways are directly related, with a correlation between the inflammatory profiles of nasal and bronchial biopsies in patients with CRSwNP.^11^ It has been reported that up to 45% of CRSwNP patients have or will develop asthma.^9^, ^12^ The prevalence of CRSwNP is higher in patients with asthma (7%) compared to the general population (4%).^13^ However, in the Finnish asthma population, the prevalence of NP has been shown to be as high as 16.5%.^7^

The mainstay therapy of CRSwNP includes medical treatments such as nasal or oral corticosteroids. For patients with CRSwNP who do not respond to conservative therapy, endoscopic sinus surgery (ESS) is considered. CRS with nasal polyps patients have been demonstrated to benefit from ESS, although a part of the CRSwNP patients have polyp regrowth and a need for a revision ESS as signs of uncontrolled disease.^14^, ^15^, ^16^, ^17^ Among Finnish patients treated with functional endoscopic sinus surgery (FESS), the prevalence of CRSwNP as the primary diagnosis has been reported to be 17%.^18^

A limited number of studies indicate that both medical interventions and FESS improve nasal outcomes in patients with CRS and asthma,^19^, ^20^ but, more information is needed about the burden of CRS and how it is affected by concomitant asthma. The aim of this nation‐wide real‐world study was to evaluate prevalence and incidence of CRSwNP in Finland, and to describe treatment burden of CRSwNP and how this is related to the presence and severity of co‐morbid asthma.

## 2 METHODS

The study was conducted with permission from the Finnish Social and Health Data Permit Authority Findata (THL/4801/14.02.00/2020 [2020/576]) by the provision of the Act on the Secondary Use of Health and Social Data (finlex 552/2019), therefore no informed consent from the patients was required.

### 2.1 Identification of patients with CRSwNP

The data was available from 1 January 2000 to 31 December 2019. The study cohort included incident adult patients with a diagnosis of CRSwNP between 1 January 2012 and 31 December 2018. The patients were identified on their index date, the date of first CRSwNP diagnosis, by having any of the following ICD‐10 diagnosis codes: J32.4 or J33.0, J33.1, J33.8, J33.9, or a polypectomy of internal nose reported by the Nordic Classification of Surgical Procedures code DHB20. Furthermore, patients were considered to have severe uncontrolled CRSwNP if they had at least two purchases of systemic corticosteroid (SCS) (ATC‐code: H02*) in 1 year and at least one revision ESS during the follow‐up. Patients with cystic fibrosis (E84*) or ciliary dyskinesia (Q33*, Q34*) were excluded from the study. The patients were identified from the national Finnish Care Register for Health Care and Register of Primary Health Care (Avohilmo) visits, from where their diagnoses, procedures, visits, and resource utilization were extracted. The data from the Care Register for Health Care was complemented with data from the Social Insurance Institution for reimbursed medications and purchases, and with data from Statistics Finland for vitality status and causes of death.

**FIGURE 1:**
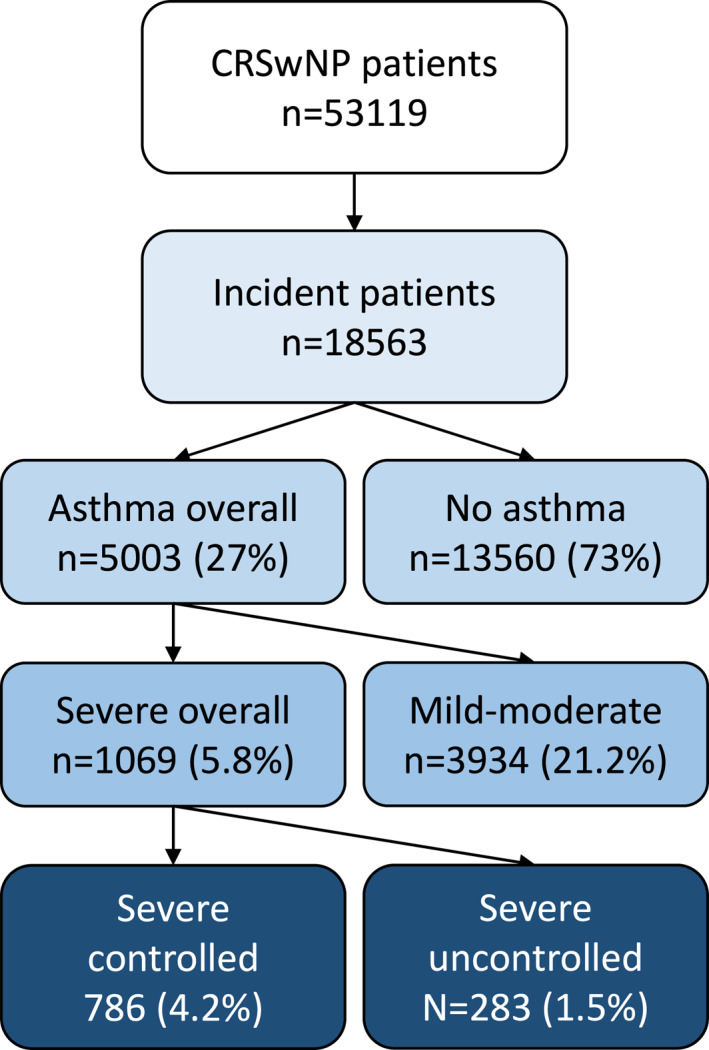
Identification of patients with chronic rhinosinusitis with nasal polyps (CRSwNP) and asthma subgroup division.

Data from 2000 to 2012 was used to assess comorbidities and clinical characteristics. The baseline period was set to 36 months prior to index, where baseline comorbidities and characteristics were described. Patients were followed up until the end of the study (31 January 2019) or death. Data for at least 1 year of follow‐up was available for all patients (Figure 1).

### 2.2 Asthma subgroups

Based on the presence and severity of asthma during baseline and follow‐up, patients with CRSwNP were divided into subgroups: no asthma, mild to moderate asthma, severe controlled asthma, and severe uncontrolled asthma. Patients were considered to have asthma if they had an ICD‐10: J45 diagnosis at least twice or at least two asthma controller medication purchases (inhaled corticosteroids [ICS] in single inhalers [ATC‐codes: R03BA01, R03BA02, R03BA05, R03BA07, R03BA08, R03BA09] or in combination with long‐acting beta‐2‐agonists [R03AK06, R03AK07, R03AK08, R03AK09, R03AK10, R03AK11]). Severe asthma was defined as asthma with a daily use of fluticasone propionate ≥800 μg or equivalent (80% adherence to 1000 μg of fluticasone propionate or equivalent) complemented with at least one other controller (Leukotriene Receptor Antagonists, Long‐acting beta‐agonist, Long‐acting muscarinic antagonists, or biologic asthma medication). The daily average ICS use was calculated as fluticasone propionate equivalent based on a sliding window of three consecutive purchases (the total micrograms of ICS at three consecutive purchases were divided by days from the first purchase to the fourth consecutive purchase). The patients with severe asthma were further divided into those with controlled and uncontrolled asthma. Asthma was considered uncontrolled in patients who had been hospitalized for asthma (ICD:10 J45‐J46), had a record of emergency room visit for asthma (ICD:10 J45‐J46), or had an outpatient visit for acute asthma (ICD‐10: J46).

Further, to assess the proportion of CRSwNP and/or asthma patients with non‐steroidal anti‐inflammatory drug‐exacerbated respiratory disease (NERD), we analyzed the number of patients with ICD‐10 code J45.* (Asthma) and/or J33.*/J32.* (Chronic sinusitis/Nasal polyps) and Z88.6 (Allergy status to analgesic agent).

### 2.3 Statistical analyses

The annual incidence and prevalence of CRSwNP were assessed during 1 January 2012–31 December 2019 by dividing the number of incident and prevalent cases by the total Finnish population. Patients who fulfilled the criteria for CRSwNP during 1 January 2000–31 December 2011 were included in the prevalence assessment. Incident patients were defined as patients with a first record fulfilling the criteria for CRSwNP during 1 January 2012–31 December 2019. The presence of a monotonic time trend in prevalence and incidence was assessed using the Mann‐Kendall test.

Patient demographics, clinical characteristics and disease burden were tabulated at baseline, overall and by subgroups, and described using summary statistics (mean with standard deviation, median with interquartile range [IQR], or frequency as the total number of patients and proportion [%] of all patients). The Charlson comorbidity index was calculated using ICD‐10 codes as implemented in the comorbidity R package.^21^, ^22^ Chi‐squared test was used for assessing differences in SCS use between the asthma subgroups during baseline.

Time to first and repeated ESS were assessed using Kaplan‐Meier fits, where time from index to surgery was defined as an event and the end of follow‐up or death as a censoring event. In the Kaplan‐Meier fit of repeated ESS, only patients with at least one ESS were included. The Kaplan‐Meier fits were visualized including the 95% Confidence Intervals (CIs), and median survival was reported, if reached. A mean cumulative count of ESS (with 95% CIs) was calculated over time overall and per subgroup using a Mean Cumulative Function.^23^

The impact of asthma (no asthma, non‐severe asthma, severe asthma, and severe uncontrolled asthma) and other concomitant diseases on CRSwNP severity (non‐severe CRSwNP and severe uncontrolled CRSwNP) were assessed using a multivariable binary logistic regression model adjusted with age as a continuous covariate and sex and the following concomitant diseases as categorical covariable: chronic obstructive pulmonary disease (identified by ICD‐10 code J44.9), diabetes (E10, E11, E14), gastroesophageal reflux disease (K21.9), hypertension (I10), obesity (E66), and thyroid dysfunction (E01‐E03).^24^ Corresponding adjusted odds ratios with 95% CIs were computed. Relative importance of the variables in the model was estimated by a global importance score based on Shapley values, which was calculated as implemented in the fastshap R package.^25^, ^26^

All statistical analyses were run using R version 4.0.3 on RStudio Server version 1.4.1103,^27^ under Microsoft Windows Server 2016 Standard.

## 3 RESULTS

### 3.1 CRSwNP prevalence and incidence

**FIGURE 2:**
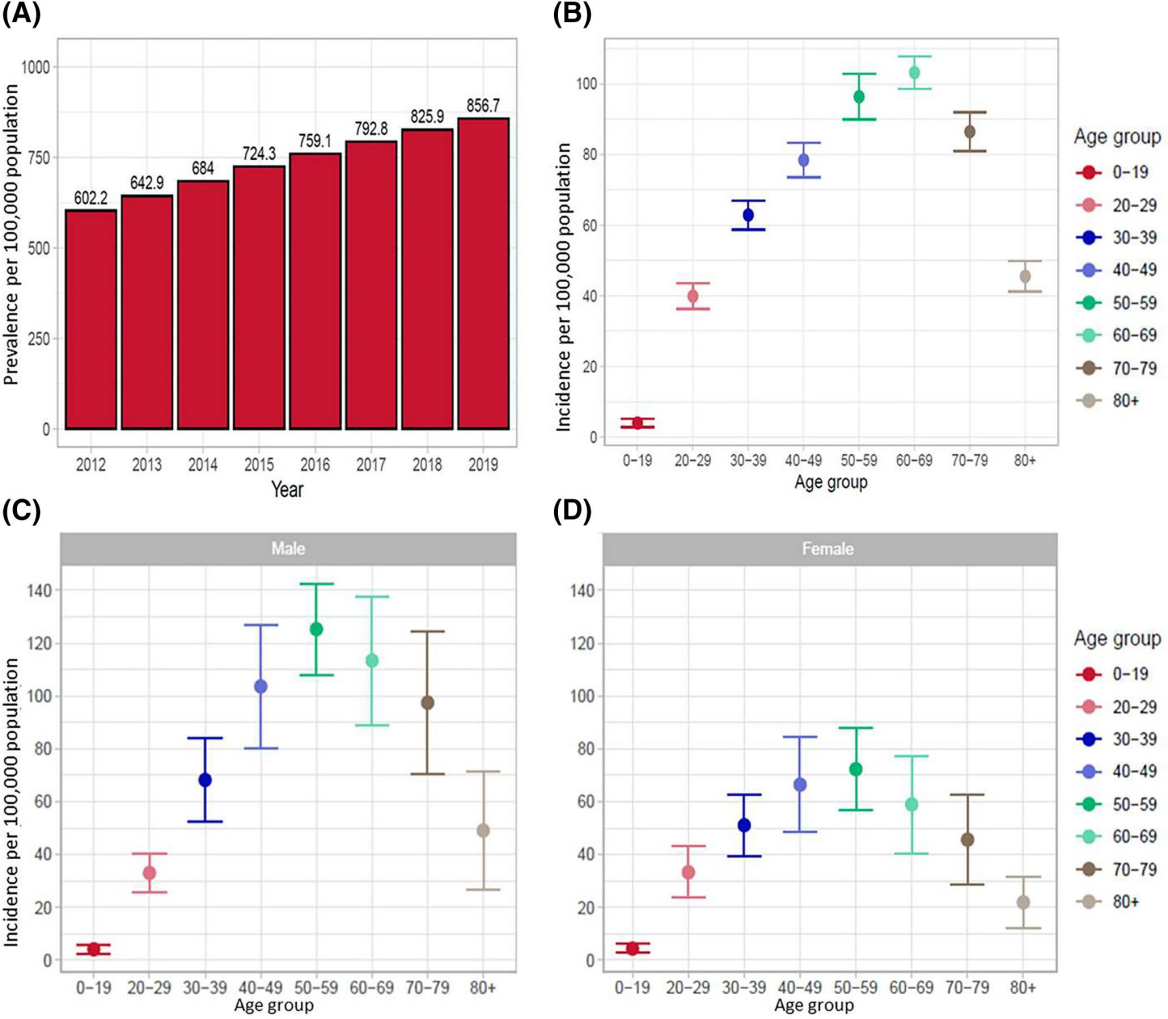
(A) Annual prevalence of chronic rhinosinusitis with nasal polyps (CRSwNP) in Finland between 2012 and 2019. Mean annual incidence of CRSwNP in Finland per age group (B) overall, (C) among males, (D) among females.

Altogether, 53,119 fulfilled the study diagnosis criteria for CRSwNP, and 18,563 (35%) were incident patients. The results show a continuous increase in lifetime prevalence of CRSwNP patients from 602.2 patients per 100,000 population in 2012 to 856.7 patients per 100,000 population in 2019 (*p* < 0.001) (Figures 1 and 2A). In the same time period, the incidence decreased from 50.5 patients per 100,0000 in 2012 to 43.4 patients per 100,000 in 2019 (*p* = 0.004).

The mean annual incidence of CRSwNP per 100,000 population are visualized in Figure 2. Patients aged 50–59 and 60–69 years had the highest incidence of CRSwNP (Figure 2B). The mean annual incidence was 96.4/100,000 population (standard deviation [SD]: 6.4) for patients aged 50–59 years and 103.2/100,000 population (SD: 4.6) for patients aged 60–69 years. Males had a higher incidence than females and the difference is highlighted in the age groups from 30 to 39 onwards (Figure 2C and 2D).

### 3.2 Demographic and clinical characteristics

Characteristics of patients with incident CRSwNP at the time of study inclusion (1 January 2012–31 December 2018) are presented in Table 1. CRS with nasal polyps patients were more frequently male (59.9%) and their median age at the time of diagnosis was 53 years (IQR 39–65). Out of all incident patients (*n* = 18,563), 27.0% had been diagnosed with asthma, with 5.8% of the incident patients having severe asthma. The number of patients with severe uncontrolled asthma was 283 patients (1.5% of the incident patients). A total of 62.7% of the patients had used any systemic corticosteroids (SCS) during the study period (2012–2019). The use of SCS was more common in patients with comorbid asthma, especially in those with more severe asthma (*p* < 0.001). Similarly, the severe uncontrolled CRSwNP was more common in patients with comorbid asthma (*p* < 0.001) with the proportion ranging from 2.4% to 16.0% in patients with no asthma to severe uncontrolled asthma, respectively. At baseline, the most common comorbidities were upper airway diseases, dental problems, cardiovascular diseases, back pain, and obesity (Supplemental Material Table S1). Nasal polyps, both acute and chronic sinusitis and acute respiratory infections were reported during the follow‐up period, Supplemental Material Table S1. Also, oral health problems, including periodontitis and dental caries persisted in patients during the follow‐up, Supplemental Material Table S2.

**TABLE 1:** Patient characteristics of the 18,563 subjects with incident chronic rhinosinusitis with nasal polyps (CRSwNP) and in subgroups of these subjects at baseline and follow‐up

	Incident patients	No asthma	Asthma				
			Asthma overall	Mild to moderate	Severe asthma		
					Severe overall	Severe controlled	Severe uncontrolled
*n*	18,563	13,560	5003	3934	1069	786	283
Male (%)	11,117 (59.9)	8548 (63.0)	2569 (51.3)	2013 (51.2)	556 (52.0)	427 (54.3)	129 (45.6)
Age (median [IQR])	53.0 [39.0, 65.0]	53.0 [38.0, 64.0]	55.0 [43.0, 65.0]	55.0 [41.0, 65.0]	56.0 [45.0, 65.0]	56.0 [47.0, 65.0]	54.0 [42.5, 64.0]
SCS (%)	11,633 (62.7)	7337 (54.1)	4296 (85.9)	3282 (83.4)	1014 (94.9)	733 (93.3)	281 (99.3)
ESS (%)	8673 (46.7)	5901 (43.5)	2772 (55.4)	2146 (54.6)	626 (58.6)	452 (57.5)	174 (61.5)
ESS once (%)	7227 (38.9)	5097 (37.6)	2130 (42.6)	1675 (42.6)	455 (42.6)	332 (42.2)	123 (43.5)
ESS repeated (%)	1446 (7.8)	804 (5.9)	642 (12.8)	471 (12.0)	171 (16.0)	120 (15.3)	51 (18.0)
Non‐severe controlled CRSwNP (%)	17,766 (95.7)	13,240 (97.6)	4526 (90.5)	3604 (91.6)	922 (86.2)	685 (87.2)	237 (83.7)
Severe uncontrolled CRSwNP (%)	797 (4.3)	320 (2.4)	477 (9.5)	330 (8.4)	147 (13.8)	101 (12.8)	46 (16.3)
Charlson comorbidity index (%)							
0	14,407 (77.6)	11,983 (88.4)	2424 (48.5)	2016 (51.2)	408 (38.2)	342 (43.5)	66 (23.3)
1	2960 (15.9)	838 (6.2)	2122 (42.4)	1564 (39.8)	558 (52.2)	376 (47.8)	182 (64.3)
2	845 (4.6)	569 (4.2)	276 (5.5)	214 (5.4)	62 (5.8)	43 (5.5)	19 (6.7)
3	253 (1.4)	114 (0.8)	139 (2.8)	107 (2.7)	32 (3.0)	19 (2.4)	13 (4.6)
4	50 (0.3)	22 (0.2)	28 (0.6)	22 (0.6)	6 (0.6)	<5 (<0.6)	<5 (1.8)
5+	48 (0.3)	34 (0.3)	14 (0.3)	11 (0.3)	<5 (<0.5)	<5 (<0.6)	<5 (1.8)

Abbreviations: CRSwNP, chronic rhinosinusitis with nasal polyps; ESS, endoscopic sinus surgery; IQR, interquartile range; SCS, any systemic corticosteroids use.

During follow up, intranasal corticosteroids purchases (ATC subgroup R01*) were found for 90% of the patients. For the baseline and follow up combined, 94.7% of patients had purchases for R01 category medications.

### 3.3 Frequency and time to first and repeated surgery in different subgroups

**FIGURE 3:**
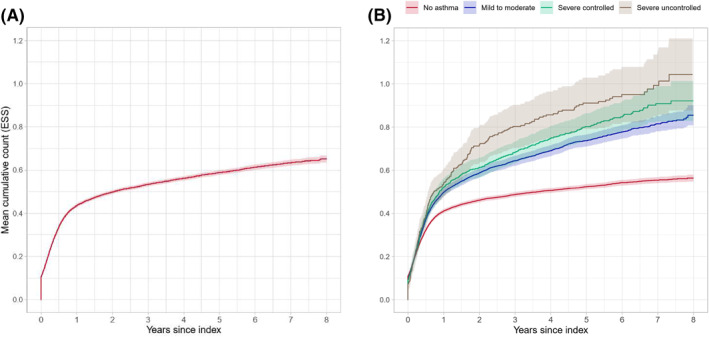
Mean cumulative count for endoscopic sinus surgery (ESS) among (A) Chronic rhinosinusitis with nasal polyps (CRSwNP) patients, and (B) by asthma status (no asthma, mild to moderate asthma, severe controlled asthma, and severe uncontrolled asthma).

The mean cumulative count with 95% CIs for ESS for all incident CRSwNP patients during an 8‐year follow‐up is presented in Figure 3A. At 1 year follow‐up, the mean number of ESS per patient was 0.44 (95% CI 0.43–0.44) ESS. At 5 years of follow‐up, the mean cumulative count per patient was 0.59 (95% CI 0.58–0.60) ESS. Both asthma status and asthma severity had an impact on the mean cumulative count for ESS, and a CRSwNP patient with severe controlled asthma had undergone on average 0.68 (95% CI 0.63–0.73) ESS within 3 years after the diagnosis. The corresponding count for a severe uncontrolled asthma patient was 0.80 (95% CI 0.70–0.90) ESS within 3 years after the diagnosis, Figure 3B.

**FIGURE 4:**
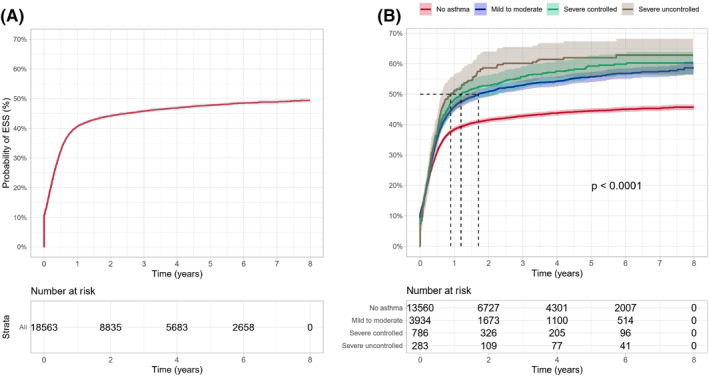
Time to first endoscopic sinus surgery (ESS) among (A) chronic rhinosinusitis with nasal polyps (CRSwNP) patients, and (B) by asthma status (no asthma, mild to moderate asthma, severe controlled asthma, and severe uncontrolled asthma).

Overall, during the follow‐up period, 8673 patients out of the total 18,563 incident CRSwNP patients (46.7%) had undergone an ESS to treat the polyps. The probability of having an ESS was 40.6% (95% CI 39.9%–41.3%) for the CRSwNP patients during the first year after the diagnosis of polyps (Figure 4A). The median time from the diagnosis of polyps to first ESS in patients with mild to moderate asthma was 20 months, while the median time for patients with severe uncontrolled asthma was 11 months. Patients in all asthma groups were more likely to have the first surgery earlier compared to non‐asthma patients. Still, for the non‐asthma CRSwNP patients, the probability of ESS was 41.5% (95% CI 40.6%–42.3%) within the first 2 years after the diagnosis of polyps, Figure 4B.

**FIGURE 5:**
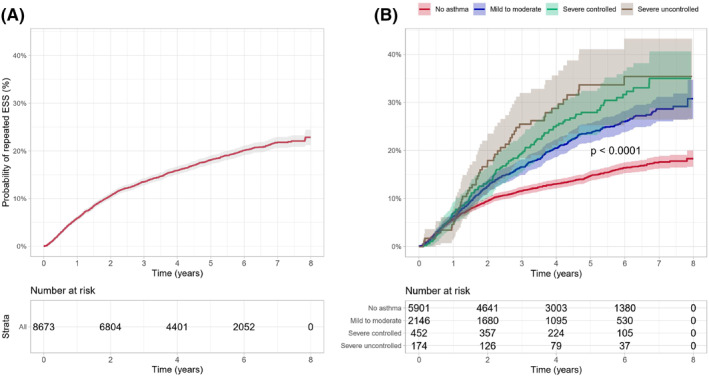
Time to repeated endoscopic sinus surgery (ESS) among (A) chronic rhinosinusitis with nasal polyps (CRSwNP) patients, and (B) by asthma status (no asthma, mild to moderate asthma, severe controlled asthma, and severe uncontrolled asthma).

Out of the 8673 CRSwNP patients who had an ESS, 7227 (83.3%) had only one ESS, whereas 1446 (16.6%) patients had two or more sinus surgeries. Overall, the probability for at least one revision ESS during follow‐up within 2 years after the first ESS was 10.6% (95% CI 10.0%–11.3%) (Figure 5A). Figure 5B shows that any asthma comorbidity increased the likelihood of having another surgery. In addition, the patients with more severe asthma were more likely to have the revision surgery earlier compared to non‐asthma CRSwNP patients. The probability of repeated ESS within 2 years after the first ESS was 9.5% (95% CI 8.7%–10.2%), 12.6% (95% CI 11.2%–14.0%), 13.5% (95% CI 10.3%–16.7%) and 17.9% (95% CI 11.9%–23.5%), for patients with no asthma, mild to moderate asthma, severe controlled asthma, and severe uncontrolled asthma, respectively.

### 3.4 Impact of asthma and other concomitant diseases on CRSwNP severity

According to the multivariable binary logistic regression model, asthma was the only disease associated with severe uncontrolled CRSwNP (*p* < 0.001) with adjusted odds ratios of 4.04 (95% CI 3.44–4.75), 7.01 (95% CI 5.50–8.93), and 8.82 (95% CI 6.25–12.43) for non‐severe, severe, and severe uncontrolled asthma, respectively. Moreover, age was negatively associated with CRSwNP severity (*p* < 0.001) with an odds ratio of 0.98 (95% CI 0.98–0.98). Asthma was a more important variable than age in the model with corresponding mean Shapley values (absolute log‐odds scale) of 0.61 and 0.40 (Supplemental Material Table S3).

### 3.5 Proportion of patients with NERD

Assessing the proportion of patients with NERD in our data, we found that none of the patients had a NERD diagnosis at baseline and only 0.3% of the patients had a NERD diagnosis during follow‐up.

## 4 DISCUSSION

In the current nationwide observational study, we found an increase in the prevalence of CRSwNP from 2012 to 2019. Further, about a quarter of the patients with CRSwNP suffered from comorbid asthma. Asthma, and especially more severe asthma, was associated with higher need for systemic corticosteroids and more frequent sinus surgeries.

In the last 2 decades, the prevalence of CRSwNP has risen,^7^, ^8^ and our study showed a continued increase during the study period, from about 6.0% in 2012 to 8.6% in 2019. The increase was particularly pronounced in the subjects between 50 and 70 years of age. There was a slight decrease in incidence of CRSwNP during the study period, however, the higher incidence at the beginning of the study period may be due to the lesser availability of data in the Avohilmo register initially, which was initiated in 2011. Thus, as the incidence remained similar throughout the study period, the slight rise in prevalence is likely related to the aging population.

The mean annual incidence peaked in patients aged 60–69 years (103.2/100,000). In general, the average age of onset of CRSwNP was 52 years, and the typical age at diagnosis ranged from 50 to 60 years, which is in line with previously reported findings.^4^, ^28^ In our study, males had a higher mean annual incidence of CRSwNP than females, albeit this is inconclusive, as there is no consensus with regards to gender in the literature.^29^, ^30^ Further, our study found that 27% of CRSwNP patients had asthma as a comorbidity, which is an estimate based on real‐world data. In examples from literature, estimates suggest that up to 67% (range, 40%–67%) of CRSwNP patients are having comorbid asthma. In a recent Finnish study analyzing the comorbidities of patients with rhinitis and rhinosinusitis, asthma was the overall most common comorbidity, especially in patients with CRSwNP, at 48.6%.^24^ The data was extracted from electronic health records of outpatients at the Helsinki and Uusimaa (HUS) Hospital District, which likely explains the difference to our findings, as the data is regional and from specialty care. Further, the reason for the difference between the findings in our study compared with previous literature may be that in real‐world, many patients with CRSwNP still have undiagnosed asthma.^31^, ^32^

In Finland, CRSwNP management is initiated rapidly after the diagnosis. About two thirds (62.7%) of the patients with CRSwNP were treated with SCSs and about half of them had ESS. In our study, more than 40% of the CRSwNP patients had ESS within the first‐year post diagnosis, 38.9% had only one ESS, whereas 7.8% needed ESS at least twice. Apart from a small study reporting a 7% revision rate in CRSwNP patients,^33^ several studies report rates of 14%–25%.^34^, ^35^, ^36^ The short time between diagnosis of CRSwNP and ESS is because the official diagnosis for CRSwNP is usually given after nasal endoscopy, which is performed at the hospital, where the patient with uncontrolled CRS has been referred for consideration of ESS.^35^

Comorbid asthma and especially severe asthma increase the probability of being treated both with SCSs and surgical treatment. In line with previous studies, our study showed that asthma status and especially the severity of asthma was associated with the likelihood of having an earlier ESS.^30^, ^32^, ^37^ In addition, asthma comorbidity increased the likelihood of repeated ESS. For patients with more severe asthma, it has been shown that CRSwNP is more difficult to manage, and that the condition increased the probability of a recurring ESS.^30^ However, ESS in CRSwNP patients is associated with a high rate of recurrence which is likely to contribute to the burden of the disease, further exacerbated by comorbidities such as asthma.^35^, ^38^

With regards to the CRSwNP patients with or without asthma, very low numbers were found to have the ICD‐10 code for NERD. This is likely explained by the fact that Z88.6 also code for any other allergic reactions due to analgetic agent, moreover, it is unfortunately rarely systematically coded by physicians. Hence the proportion of subjects with Z88.6 diagnosis was low which does not reflect the true prevalence of NERD in Finland. In the study by Nuutinen et al.,^24^ the proportion based on keyword search in clinical text was much higher, with 17.7% of all CRSwNP patients having NERD, additionally, due to more severe cases treated and better diagnostics in specialty care.

The management of patients with CRSwNP and severe uncontrolled asthma remains a challenge.^38^ There is an unmet need in improving the management of CRSwNP to achieve greater patient satisfaction and disease prevention. Targeted therapies are needed that can decrease the type‐2 inflammation common in CRSwNP and asthma, preferably as a single therapy treating both the upper and lower airway disease.^39^ In recent years, the introduction of biological therapies targeting type 2 inflammation has increased the treatment options for CRSwNP. These include dupilumab, an anti‐IL‐4R monoclonal antibody (mAb), mepolizumab, an anti‐IL‐5 mAb, and omalizumab, an anti‐IgE mAb which have now been approved for treating CRSwNP in the European Union.^40^, ^41^, ^42^ Further, benralizumab, an anti‐IL‐5R has shown to improve CRSwNP symptoms in patients with and without comorbid asthma.^43^, ^44^ Presumably, targeted biologicals may demonstrate greater beneficial effects in patients with both asthma and CRSwNP.^45^

Our results suggest that type‐2 high conditions (comorbid CRSwNP and asthma) increase probability of revision ESS. Although these findings require validation in other populations, in terms of patient counseling use, our results emphasize the importance of diagnostics and management of both CRSwNP and asthma to prevent uncontrolled disease, suffering and costs. Further, depending on the national reimbursement policies of biological medications, some patients with comorbid asthma and CRSwNP may find it easier to access these new treatments based on the severity of asthma rather than CRSwNP.

The major strength of this study is that it includes an unselected population‐based cohort of Finnish CRSwNP patients, which limit the risk of selection bias. Furthermore, it includes real‐world data from mandatory national health‐care registries with high quality and coverage from both primary and secondary care, providing a solid and unique set of data.

There are also several limitations related to this kind of retrospective database analyses. These include the risk that some information may not have been consistently recorded for all patients, potentially impacting the population size and other outcomes. Further, the asthma severity and the level of control were based on dispensed medication and health care visits without knowledge of asthma symptoms. Also, we acknowledge that the ICD‐10 code (J33.) also include antrochoanal polyps, which are rare, often unilateral, neutrophil‐rich, and usually do not reoccur after primary operation, and which have not been shown to be associated with asthma.^1^, ^46^, ^47^ Hence, if this definition bias occurred, it was of limited impact on our main results as the presence of antrochoanal polyps would have led the result toward the null association and would therefore not affect the outcome of the study, whether excluded or not. Finally, due to lack of nasal endoscopy in primary care and variation in recording practice among clinicians, false positive and negative ICD‐10 coding of CRSwNP may have been recorded, which may have affected the results. Nevertheless, patients with severe CRSwNP as well as asthmatics are usually referred to specialists for nasal endoscopy and other examinations, and thus the ICD‐10 coding of them is more accurate.

In conclusion, CRSwNP is a prevalent and increasing health problem with frequent need for treatments with potentially severe side effects. As concurrent asthma and especially severe asthma is associated with need for even more intense treatment, these subjects need special attention. New treatment modalities, such as monoclonal antibodies, are needed to tackle airway inflammation and decrease the need for systemic corticosteroids and surgical procedures to improve burden of disease in subjects with CRSwNP.

## AUTHOR CONTRIBUTIONS

Sanna Toppila‐Salmi, Kaisa Nieminen, Gunilla Telg, Lauri Lehtimäki contributed to study design. All authors Sanna Toppila‐Salmi, Juhani Aakko, Bettina Mannerström, Jenni Hällfors, Kaisa Nieminen, Gunilla Telg and Lauri Lehtimäki contributed to interpretation of data and were involved in manuscript writing and revision. Juhani Aakko and Jenni Hällfors contributed to data acquisition and analyses. All authors Sanna Toppila‐Salmi, Juhani Aakko, Bettina Mannerström, Jenni Hällfors, Kaisa Nieminen, Gunilla Telg and Lauri Lehtimäki gave final approval for manuscript publication and agreed to be accountable for all aspects of their work.

## CONFLICT OF INTEREST

Sanna Toppila‐Salmi reports a grant of GSK and consultancies for ALK Abello, AstraZeneca, ERT, Novartis, Sanofi Pharma, and Roche. All these are outside the submitted work. Lauri Lehtimäki has received fees for lectures or advisory board meetings from ALK, AstraZeneca, Boehringer‐Ingelheim, Chiesi, Circassia, GSK, Mundipharma, Novartis, Orion Pharma and Sanofi. Lauri Lehtimäki owns shares of Ausculthing Oy. Gunilla Telg and Kaisa Nieminen are employees of AstraZeneca. The remaining authors has no conflicts to declare.

## Supporting information

Table S1

Table S2

Table S3
